# Autophagy-lysosome pathway alteration in ocular surface manifestations in Fabry disease patients

**DOI:** 10.1186/s13023-022-02441-3

**Published:** 2022-07-23

**Authors:** Marco Marenco, Marco Segatto, Marta Sacchetti, Pietro Mangiantini, Francesca Giovannetti, Rocco Plateroti

**Affiliations:** 1 Ophthalmology, Department of Sense Organs, University Sapienza of Rome, Viale del Policlinico 155, 00161 Rome, Italy; 2 Department of Biosciences and Territory, University of Molise, 86090 Pesche, Isernia Italy

**Keywords:** Fabry disease, Lysosomal storage disorder, Cornea, Autophagy-lysosome pathway, LC3 protein

## Abstract

**Background:**

Fabry disease (FD) is a rare X-linked, lysosomal storage disorder caused by mutations in the alpha-galactosidase gene and characterized by neurological, cutaneous, renal, cardiovascular, cochleo-vestibular and ocular manifestations. The aim of this study is to characterize morphological, functional and autophagy-lysosome pathway alterations of the ocular surface in FD patients.

**Methods:**

Eleven subjects with a diagnosis of FD and fifteen healthy control subjects were examined. All patients underwent ocular surface slit lamp examination, corneal aesthesiometry and in vivo confocal laser-scanning microscopy (CCM). Conjunctival impression cytology was performed in six FD patients and six controls, to assess for expression of two markers of the autophagy-lysosome pathway: the microtubule-associated protein light chain 3 (LC3) and lysosome-associated membrane protein 2 (LAMP2).

**Results:**

Cornea verticillata and increased conjunctival vessel tortuosity were detected respectively in 67% and 33% of patients with FD. Compared with healthy subjects, patients affected by FD showed a significant reduction in corneal nerve fiber length, density and nerve branching on CCM and a significantly increased expression of LC3 on conjunctival impression cytology (*p* < 0.001). No changes were observed in the conjunctival expression of LAMP2 between the two groups.

**Conclusions:**

This study shows that FD is associated with ocular surface alterations including corneal and conjunctival morphology, innervation and vascularization changes. Our data demonstrate an increased expression of LC3 protein in patients with FD, suggesting that alteration of the autophagy-lysosome pathway may play a role in the occurrence of ocular manifestations.

## Background

Fabry disease (FD) is a rare, X-linked, lysosomal storage disorder characterized by total or partial functional deficiency of the enzyme α-galactosidase A, caused by mutations in the α-galactosidase A gene (GLA). A reduction or absence of activity of this enzyme determines an altered metabolism of glycosphingolipids, such as Globotriaosylceramide (Gb3) [1–3] and galabiosilceramide [1], and progressive accumulation of these molecules in several organs including heart, kidney, eyes, brain and skin [1–5]. Patients may develop renal failure, cardiomyopathy and cerebrovascular disease, leading to a short life expectancy [6]. According to data from the Fabry Registry (2008), affected patients have a shorter life expectancy compared to the general population in the United States (58.2 years vs. 74.7 years for males, 75.4 years vs 80.0 years for females), with cardiovascular disease being the most common cause of death [7].

The estimated incidence in the United States is between 1:40,000 and 1:117,000 live births for males [6, 8, 9].

Ocular manifestations are observed in most FD patients, and involve the cornea, conjunctiva, lens and retina [10–13]. The most frequent ocular sign, and sometimes the only sign in heterozygous females, is cornea verticillata, a yellow–brown whorl-like opacity at the inferior part of the cornea caused by the storage of glycolipids within the basal corneal epithelial cells [3, 4, 14–17]. Conjunctival and retinal vessels also exhibit an increased tortuosity and aneurysmal dilatations caused by accumulation of glycosphingolipids in the endothelial cells [2, 18–21]. Less frequently, patients with FD also present a typical lens opacity with a “spoke-like” pattern at the level of the posterior capsule, usually called “Fabry cataract”; another type of lens opacity that may develop at the level of the anterior capsule is characterized by wedge-shaped white granular deposits [3, 15]. Other ocular manifestations, such as angiokeratomas of the eyelid, periorbital edema, papilledema, optical atrophy [1, 3, 5], and damage of the corneal nerves have been described [22, 23].

Currently there is on average a 15-year delay in the diagnosis of FD, due to the lack of awareness about the disease and non-specific symptoms [6, 24]. The diagnosis is based on a thorough family history, on the evaluation of enzymatic activity of alpha-galactosidase A on leukocytes and plasma [25]; the evaluation of Gb3 levels in serum and urine [26], and on cardiac and renal biopsies [2]. Therefore, novel, non-invasive, early biomarkers of FD are highly sought-after, in order to increase our understanding of the mechanisms underlying FD and to improve the management of this challenging condition. An involvement of the autophagy-lysosome pathway in the development of multisystemic damage in FD, has been recently described [27]. Nelson et al. reported widespread alterations in the autophagy-lysosome pathway associated to neurodegenerative phenotype in the brain of a mouse model of alpha-galactosidase A deficiency: notably, microtubule-associated protein light chain 3 (LC3), a marker of autophagic vacuoles, was substantially increased in multiple brain regions, and likewise, lysosome-associated membrane protein LAMP-1, a lysosomal marker, was increased also in vascular endothelial cells, suggesting an aberrant autophagy process in FD [27].

Chung et al. observed that the altered autophagy typically found in this disease could play a role in renal tubulointerstitial fibrosis and suggest that this pathway should be studied as a new therapeutic target in FD [28]. This study aims to characterize ocular surface alterations in patients with FD including conjunctival changes of autophagy pathway in order to identify potential, non-invasive biomarkers of FD.

## Methods

Eleven patients with clinical and biochemical diagnosis of FD, confirmed by genetic tests showing mutations on the GLA gene were included in the study (six males and five females; mean age 45,8 ± 18,7 years). In addition, 15 healthy, sex and age matched subjects were included as a control group. Patients who were contact lens users, and/or who were affected by ocular or systemic diseases which may influence clinical and biological examinations, and/or who were in treatment with topical ophthalmic drugs were excluded from the study.

All subjects underwent the following ophthalmological examination: (i) slit lamp biomicroscopy to detect anterior segment abnormalities including the presence of cornea verticillata and conjunctival vessels tortuosity, (ii) assessment of corneal sensitivity by Cochet-Bonnet aesthesiometer [29], and (iii) confocal in vivo laser-scanning microscopy to evaluate changes in corneal innervation and reflectivity at the basal epithelial cells layer.

Conjunctival impression cytology was also performed in six FD patients and in six controls to evaluate the expression of LC3 and lysosome-associated membrane proteins (LAMP) 2 proteins.

### Confocal laser-scanning microscopy (CCM)

All subjects underwent examination with a CCM (Rostock Corneal Module/Heidelberg Retina Tomograph III, Heidelberg Engineering, Germany). One eye of each FD patient and healthy subject, was randomly selected for examination and anesthetized with one drop of oxybuprocaine hydrochloride 0.4%. A large drop of gel was applied to the tip of the lens, and another small amount of gel was applied to the front surface of the Tomocap; the instrument was aligned with the center of the cornea, and advanced forward until it touched the cornea.

The entire corneal epithelium was scanned in order to find an increased reflectivity of the layers, suggesting the presence of Gb3 deposits; going even deeper, the subepithelial corneal nerve plexus was evaluated. The images obtained were evaluated by a masked operator (P.M.) who selected the most representative images of the plexus of each subject and evaluated using NeuronJ (ImageScience): (1) the corneal nerve fiber length (NFL), calculated as the total length of all nerve fibers and branches per scan area (mm^−1^); (2) the corneal nerve fiber density (NFD), measured as the total number of major nerve fibers per scan area (mm^−2^); and (3) the corneal nerve branch density (NBD), calculated as the number of branches emanating from all major nerve trunks per scan area (mm^−2^).

### Conjunctival impression cytology

Conjunctival impression cytology was performed in six FD patients and in the control group, by applying a methylcellulose disk to the bulbar temporal and nasal conjunctiva, after instillation of topical oxybuprocaine hydrochloride 0.4% [30].

The collected samples were evaluated by immunohistochemistry to investigate the expression of LC3 and LAMP2 proteins.

Briefly, samples were hydrated in phosphate buffered saline (PBS), and the endogenous peroxidase block was performed by incubating the cells for 3 min with 3% hydrogen peroxide. They were subsequently incubated for one hour at room temperature with goat serum (NGS) diluted in 10% PBS, and finally incubated overnight at 4 °C with the primary antibodies (LC3, Santa Cruz, sc-28266; LAMP2, Santa Cruz, sc-18822). The following day, they were incubated sequentially with a secondary anti-rabbit antibody conjugated to horseradish peroxidase (HRP) and with a streptavidin / peroxidase complex (Vectastain Universal QUICK HRP R.T.U., Vector). The immune complexes were visualized through ImmPACT DAB (Vector). The electronic images were acquired through bright field microscopy and collected in CS5 Adobe Photoshop format. The densitometries related to the immunopositivity of the photographed fields were made through the ImageJ Software for Windows program.

### Statistics

Quantitative variables were expressed as mean and standard deviation. The qualitative variables were expressed as percentages. To verify the existence of statistically significant differences in measured values of corneal sensitivity, corneal nerve morphology parameters and LC3 and LAMP2 protein expression between patients with FD and healthy subjects, an independent samples t-test was used. Spearman-rho test was used to evaluate the correlation between the variation of nerve morphology parameters evaluated and corneal sensitivity values. *P*-value < 0.05 was considered statistically significant.

## Results

**Fig. 1:**
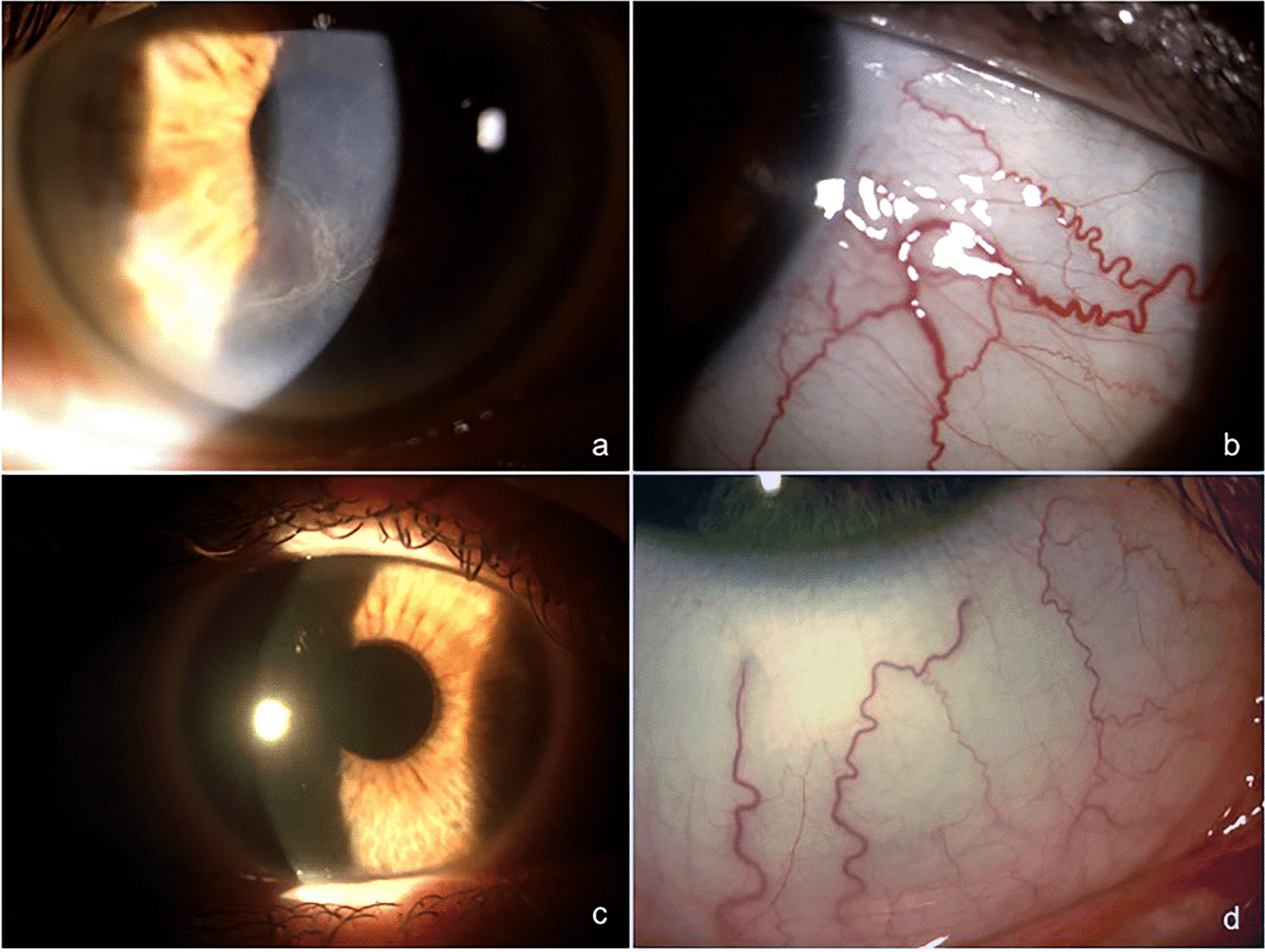
“Cornea verticillata and conjunctival vessels tortuosity” Slit lamp bio-microscopy image of a patient with FD showing the presence of cornea verticillata (**a**), a yellow–brown, whorl-like opacity caused by the storage of glycolipids within the basal corneal epithelial cells, and the increased tortuosity and aneurysmal dilatations of conjunctival vessels caused by accumulation of glycosphingolipids in the endothelial cells (**b**). Healthy cornea (**c**) and conjunctival vessels (**d**) of a control subject

Eight patients (70%) with FD showed cornea verticillata. Four patients (35%) with FD showed increased conjunctival vessels tortuosity in the inferior part of bulbar conjunctiva at slit lamp examination. (Fig. 1). Patients with FD showed significant decrease of corneal sensitivity when compared with healthy controls (5.58 ± 0.30 vs 5.92 ± 0.13 cm, *p* = 0.0325).

**Fig. 2:**
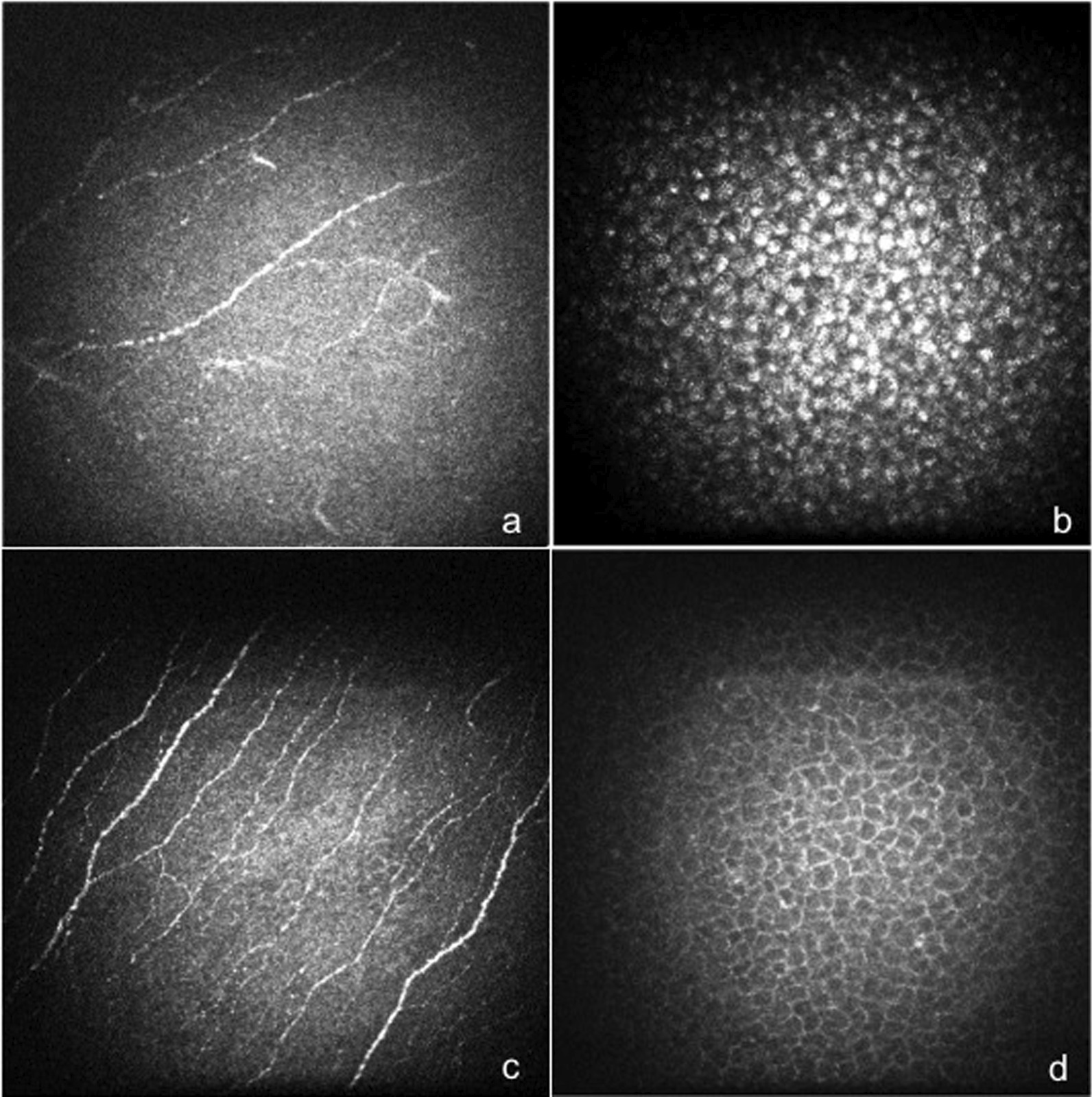
“In vivo corneal confocal microscopy in Fabry Disease” In vivo corneal confocal microscopy examination showed significant decrease of NFL, NFD and NBD of the sub-basal nervous plexus (**a**) and an increase of reflectivity at the basal cells layer of the corneal epithelium due to intracellular highly reflective irregular material (**b**) in a patient with FD. Normal sub-basal nervous plexus (**c**) and normal reflectivity of the basal cells layer of corneal epithelium (**d**) of a healthy subject

The in vivo corneal confocal microscopy examination showed an increase of reflectivity at the basal cells layer of the corneal epithelium in all FD patients when compared with the control group in 18% of patients with FD. Patients with FD showed significant decrease of NFL, NFD and NBD when compared with healthy subjects (NFL: FD: 8,53 ± 2,94 vs healthy controls 11,84 ± 3.73 mm-1, *p* = 0.040; NFD: FD: 25 ± 10.46 vs healthy controls 52.78 ± 20,98 fibers/mm2, *p* = 0.001; NBD: FD: 13,07 ± 8.59 vs healthy controls 40,28 ± 17,43 branches/mm2, *p* < 0.001) (Fig. 2).

Although corneal sensitivity values measured by Cochet Bonnet aesthesiometer did not significantly correlate with NFL values (*p* = 0.02, R = 0.74), changes of corneal nerve morphology were significantly correlated with the decrease of corneal sensitivity: NFD (*p* < 0.001, R = 1,00), NBD (*p* = 0.0003, R = 0.99).

**Fig. 3:**
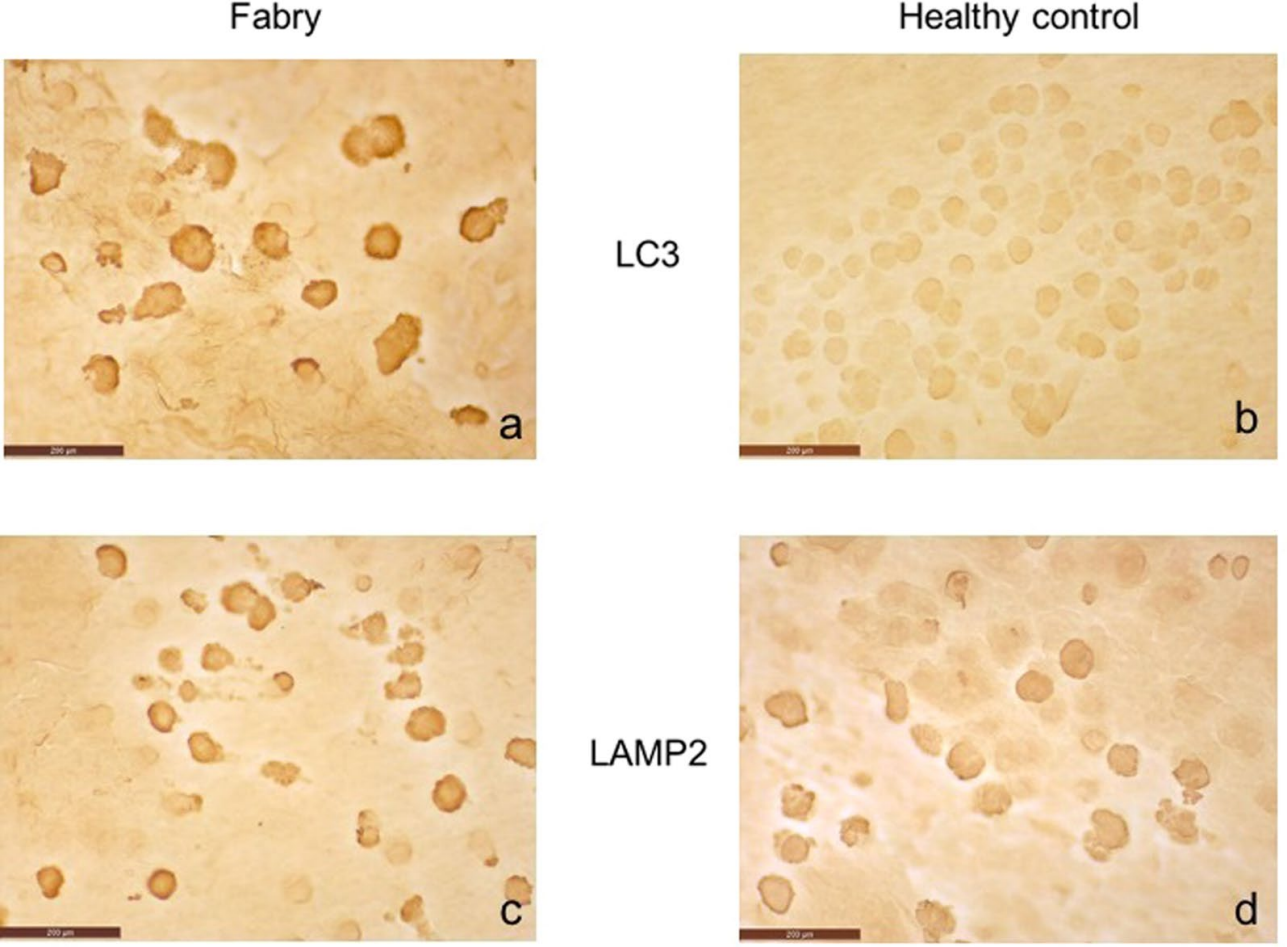
“LC3 and LAMP2 expression on conjunctival epithelium” Immunohistochemistry of conjunctival impression cytology samples identified a statistically significant increase in mean expression of LC3 in FD group (**a**) when compared with healthy control group (**b**), while no statistically significant difference were observed in mean expression of LAMP2 between FD (**c**) healthy subjects (**d**)

Immunohistochemistry of conjunctival impression cytology samples identified a statistically significant increase in mean expression of LC3 between the FD group and the control group (*p* < 0.001). No statistically significant difference in the expression of LAMP2 was detected (Fig. 3).

## Discussion

The aim of this study was to characterize the ocular surface alterations in FD patients and to address the involvement of the autophagy-lysosome pathway alterations.

Our study confirmed the high frequency of clinical ocular alterations in FD such as cornea verticillata (70%) and increased of conjunctival vessels tortuosity (35%). Currently, cornea verticillata is considered the ocular hallmark of FD. The observation of an increased conjunctival vessels tortuosity, mainly located at the inferior part of bulbar conjunctiva, in 35% of FD patients is in line with previous data and related to the progressive accumulation of Gb3 in endothelial cells [18–21].

Our data also showed that approximately 20% of FD patients had an increased reflectivity of the basal cells layer of corneal epithelium, as detected by CCM, suggesting a possible employment of this diagnostic technique to improve the identification of the Gb3 deposits [4, 5, 16, 17]. Moreover, CCM showed marked alteration of the subepithelial corneal nerve plexus in patients with FD. Interestingly, the changes of corneal nerve morphology correlated with an impairment of corneal sensitivity, supporting the hypothesis to use these techniques to perform an early diagnosis of small-fiber neuropathy in FD patients, as suggested for diabetic neuropathy [22, 23].

Immunohistochemistry performed on conjunctival impression cytology samples showed increased levels of LC3 protein in FD patients when compared with the control group.

An increased expression of autophagosome LC3 protein, due to impaired autophagic function, is a common observation in lysosomal storage disorders, such as Fabry disease, as demonstrated by immunohistochemistry performed on renal biopsy samples [27]. The autophagic vacuoles contain undegraded material accumulated within the cells. This material, that has a lamellar appearance, appears to be similar to the one found in other cell lines of patients suffering from Fabry disease [31]. In this study, we observed an increased expression of LC3 in the conjunctival epithelium of FD patients, confirming the hypothesis of an increased number of autophagic vacuoles in conjunctival cells, analogously to those found in renal cells of FD patients.

Our results did not show significant changes of conjunctival levels of LAMP2, although an increase in the expression of this protein in phagocytes of Fabry patients has been previously demonstrated [32]. This contrasting data suggest that the autophagic and lysosomal pathways of conjunctival epithelial cells are quite different from other cell types. Notably, it is possible to hypothesize that the LAMP2 turnover could be slower, and the protein levels more stable, leading to difficult detection of protein variation in conjunctival epithelium.

## Conclusion

This study highlights the marked alteration of the ocular surface in FD patients and suggests to further evaluate the clinical use of CCM and conjunctival impression cytology, two simple/non-invasive diagnostic techniques, to monitor Fabry disease progression and response to treatment.

## Data Availability

The datasets used and/or analysed during the current study are available from the corresponding author on reasonable request.

## Abbreviations

FD: Fabry disease
CCM: Confocal laser-scanning microscopy
LC3: (Microtubule-associated protein) light chain 3
LAMP2: Lysosome-associated membrane protein 2
GLA: α-Galactosidase A gene
Gb3: Globotriaosylceramide
NFL: Nerve fiber length
NFD: Nerve fiber density
NBD: Nerve branching density
